# Effects of concurrent caffeine and mobile phone exposure on local target probability processing in the human brain

**DOI:** 10.1038/srep14434

**Published:** 2015-09-23

**Authors:** Attila Trunk, Gábor Stefanics, Norbert Zentai, Ivett Bacskay, Attila Felinger, György Thuróczy, István Hernádi

**Affiliations:** 1 Department of Experimental Neurobiology, University of Pécs, Hungary; 2 Translational Neuromodeling Unit (TNU), Institute for Biomedical Engineering, University of Zurich & ETH Zurich, Switzerland; 3 Laboratory for Social and Neural Systems Research, Department of Economics, University of Zürich, Switzerland; 4 Department of Analytical and Environmental Chemistry, University of Pécs, Hungary; 5 Szentágothai Research Centre, University of Pécs, Hungary; 6 National Institute for Radiobiology and Radiohygiene (NIRR), Budapest, Hungary

## Abstract

Millions of people use mobile phones (MP) while drinking coffee or other caffeine containing beverages. Little is known about the potential combined effects of MP irradiation and caffeine on cognitive functions. Here we investigated whether caffeine intake and concurrent exposure to Universal Mobile Telecommunications System (UMTS) MP-like irradiation may interactively influence neuro-cognitive function in an active visual oddball paradigm. In a full factorial experimental design, 25 participants performed a simple visual target detection task while reaction time (RT) and electroencephalogram (EEG) was recorded. Target trials were divided into Low and High probability sets based on target-to-target distance. We analyzed single trial RT and alpha-band power (amplitude) in the pre-target interval. We found that RT was shorter in High vs. Low local probability trials, and caffeine further shortened RT in High probability trials relative to the baseline condition suggesting that caffeine improves the efficiency of implicit short-term memory. Caffeine also decreased pre-target alpha amplitude resulting in higher arousal level. Furthermore, pre-target gamma power positively correlated with RT, which may have facilitated target detection. However, in the present pharmacologically validated study UMTS exposure either alone or in combination with caffeine did not alter RT or pre-stimulus oscillatory brain activity.

Millions of people routinely use handheld mobile phones (MP). Most of the energy of electromagnetic fields (EMF) emitted by MPs is absorbed in the head of the user and may affect cognitive functions^1^. People often use EMFs emitted by MPs and consume stimulants (e.g., caffeine) at the same time without awareness of possible combined effects^2^. Evidences indicate that the combination of caffeine and other EMFs, such as light, may alter arousal levels and cognitive functions^3^,^4^. However, to date, most available research on human cognition have only investigated the effects of different types of MP exposures or caffeine alone without considering their possible additive effects^1^,^2^,^5^.

It is well known that caffeine exerts facilitatory effects on human cognition^6^,^7^,^8^,^9^,^10^,^11^,^12^, which are thought to be indirectly brought about by altering calcium channel activation^13^ via blocking natural inhibitory effects mediated by adenosine A1/A2 receptors^14^. Weak EMFs have also been reported to alter intracellular signaling by increasing calcium ion permeability of the cell membrane^15^,^16^ or altering the expression of calcium binding proteins^17^,^18^,^19^. While calcium plays an important role in cognitive functions^20^,^21^,^22^, any combined effects of caffeine and MP exposure on calcium related mechanisms may affect cognitive performance indexed by reaction time and brain oscillatory activity.

In the present study we focus on the effects of caffeine and MP exposure on cognitive information processing indexed by electroencephalographic (EEG) measures of brain function in correlation with behavioral measures of reaction time (RT). Here we focus on analyses of pre-target oscillatory activity in the alpha and gamma frequency bands as they are considered to be neuronal signatures of stimulus processing and the functional basis of perception and cognition^23^.

First, we tested the possible combined effects of caffeine and MP exposure on the pre-target alpha band. Numerous studies investigated the effects of caffeine on brain activity in the alpha band. Most of them reported that alpha activity is affected by caffeine, namely caffeine decreases the power of resting state alpha band indicating increased actual arousal state^6^,^11^,^24^,^25^. Several other studies suggested that weak EMFs emitted by MPs may also alter brain oscillatory activities especially in the alpha band^1^,^26^,^27^. Alpha band itself plays an important role in different mechanisms such as active inhibitory mechanisms^28^ or task-dependent cortical processing^29^ as well. This frequency band, particularly in the pre-target period, is one of the possible determinants of top-down processing which enhances the speed of sensory input detection^30^,^31^.

Second, we measured the possible combined effects on the gamma band activity. Oscillatory activity in the gamma frequency band is known to facilitate stimulus processing as well^32^. Several studies suggested that gamma oscillations play key roles in attention and stimulus expectation. While attention to a stimulus increases the amplitude of gamma activity, the expectation of a stimulus decreases it^23^,^33^,^34^. Several studies showed the role of pre-target gamma activity in determining the speed of RT. For example, positive correlation was found between pre-target gamma power and RT^35^, showing that lower gamma power was associated with faster RT. Thus, the changes of gamma activity in the pre-target (expectation) period may facilitate the processing of the forthcoming target event^36^.

Here we analyzed the recorded data in conjunction with a previous study^5^ in a different aspect. In a previous paper we analyzed the potential effects of caffeine and EMF on stimulus-evoked brain potentials (P300). Here we focus on spectral power of pre-target oscillatory activity because several studies found that caffeine and EMF alters brain oscillations. In the current study, we aimed at investigating the potential effects of caffeine and UMTS MP exposure on the different local probabilities of the target stimuli indexed by RT and pre-target brain oscillations. Specifically, we investigated how RT and pre-target alpha and gamma spectral amplitude in different local target probability categories may be affected by caffeine, MP exposure or the combination of these two factors.

Our hypothesis was that, due to previously reported^1^,^6^ similar facilitatory effects on brain excitatory activity, simultaneous caffeine and MP exposure will have a larger effect than caffeine or MP EMF exposure alone.

## Materials and Methods

### Participants

Twenty-five healthy, right-handed, non-smoker university students [9 female, age range 18 to 38 years, mean 21.07, standard deviation (SD) 3.58] participated in the study, who regularly consume 1–2 cups of tea/coffee by self-report. Because the half-life of caffeine in the body is reduced by 30 to 50% in smokers compared to nonsmokers^13^, here we enrolled only nonsmokers. Participants were asked to abstain from any kind of caffeine-containing substances and alcohol at least 10 and 24 hour prior to each session, respectively. All participants gave their written informed consent after the nature of the experiment had been fully explained. The study was conducted according to the ethical principles stated in the Declaration of Helsinki and applicable national guidelines. The protocol of the study was approved by the Ethical Committee of the University of Pécs. Written informed consent was obtained from all volunteers. EEG recordings were carried out at the Psychophysiology Laboratory of the Integrative and Translational Neuroscience Research Group at the University of Pécs, Hungary.

### Caffeine concentration measurement from saliva samples

Saliva samples were taken at the beginning and the end of each recording session and caffeine concentrations were determined by high-performance liquid chromatography (HPLC). Raw saliva samples were centrifuged for 20 min at 4000 rpm and at 4 °C. About 1.5 to 2 ml supernatants were centrifuged again at 13000 rpm and at 24 °C. About 0.5 to 1 ml of the supernatant was stored at −80 °C for later HPLC analysis. (For the details about the HPLC analysis, see supplementary data in our previous study by Trunk *et al.*^5^).

### Caffeine treatment

Three mg/kg caffeine packed in identical hard gelatin capsules were administered to the participants. The capsules were administered per os with 200 ml still mineral water. We used 5, 10, 20, and 100 mg caffeine-filled capsules. The average body weight was 70.52 kg (SEM: 3.66) and the average caffeine dose was 211.56 mg (SEM: 10.98). For placebo treatment, glucose filled gelatin capsules were used. Placebo capsules contained less than 0.5 g glucose per capsule without any additional substance. Similar capsules were used for each treatment. To avoid possible influences caused by subjective bias on the number of capsules taken, volunteers received the same amount of capsules in the control (placebo) sessions as in the caffeine sessions.

### EEG recording

EEG was recorded with a 32-channel BrainAmp amplifier (Brain Products GmbH, Munich, Germany) using silver-silver-chloride (Ag/AgCl) electrodes placed according to the International 10–20 system in an elastic cap (Easycap, Munich, Germany). The nose served as reference and the forehead as ground. An additional electrooculography (EOG) electrode was placed above the right external canthus. The impedance was measured at the beginning of each session and was adjusted to less than 5 kOhm at all electrodes. On-line band-pass filters were used between 0.016 Hz and 450 Hz with an additional notch filter to attenuate power line at 50 Hz. Raw data were digitized at 16 bit at a sampling rate of 1 kHz. Participants were asked to keep their head and eye-movements at minimum during the whole recording session.

### UMTS exposure device

**Figure 1:**
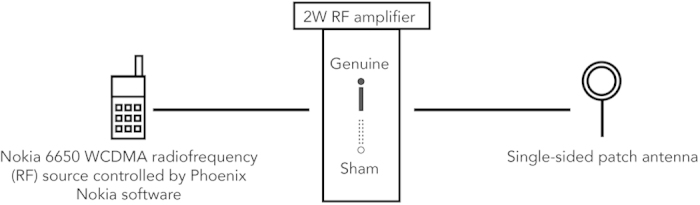
Schematic drawing of the exposure system. During the whole EEG recording session the patch antenna was unilaterally placed at a distance of 4 to 5 mm from the right ear above the tragus, mimicking the most frequent normal position of MP in use as reported by the participants. The phone was connected to a 2W RF amplifier and controlled by the Phoenix Service Software (Nokia).

The UMTS MP exposure system was previously developed and successfully used in previous studies^2^,^5^,^37^,^38^. The UMTS radiofrequency (RF) exposure was administered by means of a standard Nokia 6650 (Nokia, Espoo, Finland) MP via Phoenix Service Software (v. 2005/44_4_120; Nokia, Espoo, Finland) for 15 minutes (Fig. 1). The MP was connected to an external patch antenna, which was mounted on a plastic headset. Double-blind experimental conditions were ensured by a two-position switch (A or B) located on the front panel of the RF amplifier: one position was associated with genuine exposure, and the other with sham exposure. The investigator was not aware of the actual exposure condition. The peak SAR averaged on 1 g tissue was 1.75 W/kg^38^ at 2 cm depth from the shell surface of the phantom, and the averaged SAR over 10 g was set below 2 W/kg in any position within the phantom. These values were below the 2 W/kg limit for RF exposure of the general public as requested by the 1999/519/EC Recommendation. (For the details on the exposure device and conditions, see supplementary data in our previous study by Trunk *et al.*^5^).

### Stimuli and procedure

**Figure 2:**
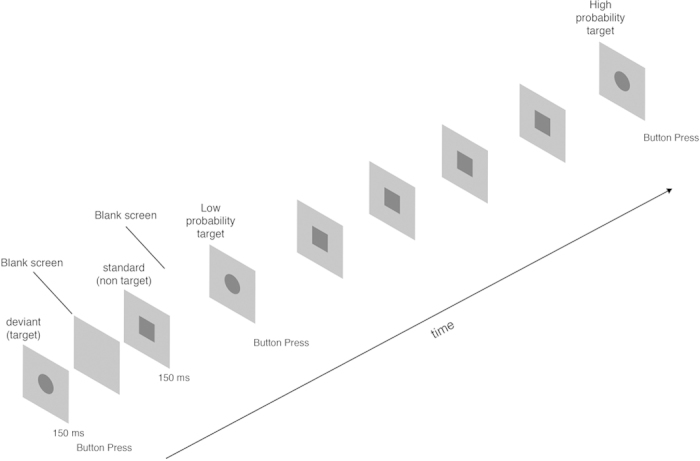
Schematic illustration of the experimental design. In each session dark grey squares were presented as frequent standard (p = 0.8) and a circles as rare deviant (p = 0.2) stimuli on a light grey background. The participants’ task was to press a button on each occurrence of the rare stimulus. Reaction time and pre-target EEG activity to the target stimuli were sorted by target-target. For the probability analysis we defined Low and High probability categories. In the Low probability category and in the High probability category 1 and 4 standard stimuli preceded the target, respectively. Stimulus-onset asynchrony was randomized between 1000–2000 ms.

In a double blind, crossover experimental design, the participants took part in four experimental sessions, corresponding to the four possible exposure conditions (Control—placebo caffeine & sham UMTS, UMTS—placebo caffeine & genuine UMTS, Caffeine—genuine caffeine & sham UMTS and Combined—genuine caffeine & genuine UMTS). In the visual oddball task, a square as frequent standard (p = 0.8) or a circle as rare deviant (p = 0.2) were presented in a pseudorandom order (Fig. 2). The trial numbers for standard and deviant stimuli were 640 and 160, respectively. Each recording session consisted of three consecutive recording blocks or trials [2.5 min pre exposure block (standard trials: 80; deviant trials: 20), 15 min genuine or sham MP exposure block (standard trials: 480; deviant trials: 120), 2.5 min post exposure block (standard trials: 80; deviant trials: 20)] with no breaks between blocks. During the whole session the patch antenna was unilaterally placed at a distance of 4 to 5 mm from the right ear above the tragus, mimicking the natural position of MP during a call. The stimulus-onset asynchrony (SOA) varied between 1000 and 2000 ms.

### Data analysis

Behavioral and EEG data were analyzed off-line on a personal computer using built-in, self-developed scripts and freeware EEGLAB toolbox^39^ in the Matlab (MathWorks, Natick, MA) programming environment. To test for the possible acute interaction effects of caffeine and MP exposure on reaction time and EEG we analyzed data from the exposure block.

**Figure 3:**
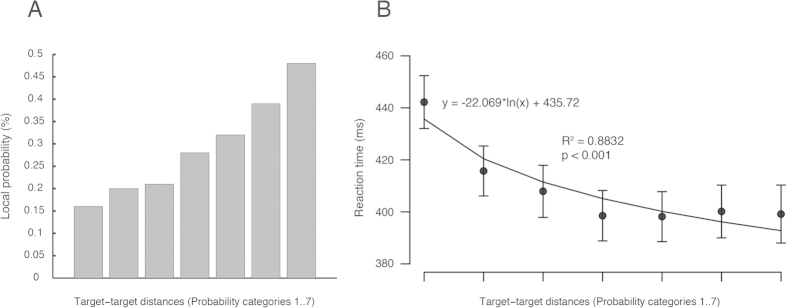
(**A**) Results for local probabilities in each probability category. The probability of the target as a forthcoming stimulus increases after each standard stimulus is presented before the target. Here, 90% of the stimuli were presented in probability categories 1 to 7. Ten percent of the targets, which were preceded by more than 7 standards (8 to 14), were not analyzed here. (**B**) Results for reaction time (RT) to target stimuli in each probability category. The y = a*log(x) + b linear-log statistical model revealed significant (p < 0.001) logarithmic correlation (R^2^ = 0.8832) between the target-target distances (target probabilities) and the reaction times. Kendall’s test showed marginal correlation (tau = −0.619, p = 0.069) on the RTs across the probability categories (1 to 7), with shorter RT in higher probability categories. Furthermore, we found significant difference between High and Low probability categories. Note: for abbreviations see Fig. 2.

Reaction time and EEG amplitude in the 600 ms interval preceding target onset were binned based on target-target distances. The procedure resulted in 7 different stimulus categories according to target local probability (Prob) from category 1 to category 7. The local probabilities of these categories in the stimulus sequence were calculated in all conditions (Control, UMTS, Caffeine and UMTS) with the following formula:


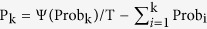


where T is the number of trials in the analyzed block (T = 120), Ψ counts the number of the targets in the actual (k) Prob category and i increments in each cycle. For example, if 22 Prob1 trials are located in the sequence [Ψ(Prob_1_) = 22] then the local probability of the Prob1 is 22/120 = 0.18. If Ψ(Prob_2_) = 26, then the local probability of the Prob2 is 26/(120 − 22) = 0.22. The local probability of the Prob3 is Ψ(Prob_3_)/[120 − (22 + 26)], etc. Figure 3a shows the probabilities of each Prob category.

To study treatment effects on sequential stimulus processing, we used a modified version of data separation method by Holm *et al.*^40^. Data from Prob1 trials where only one standard stimulus preceded the target were assigned to the low probability (Low) category while data from Prob4 trials where four standard stimuli preceded the target were assigned to the high probability category (High) and were selected for further analysis. We used the terms of Low and High because prior studies have shown no difference between the RTs to targets if the number of the preceding standard stimuli were more than four^41^. Thus Low (Prob1) and High (Prob4) can be considered as most representative categories to describe the possible involvement of different target expectancy levels in task performance.

To reveal potential interactions of caffeine and UMTS MP exposure in RT and oscillatory measures we focused on the exposure block and we used the additive analysis model with the following formula, as applied elsewhere^42^,^43^,^44^:


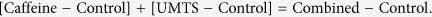


Hereafter, “[Caffeine − Control] + [UMTS − Control]” and “Combined − Control” are referred to as ‘sum’ and ‘simultaneous’ data, respectively. We hypothesized that violation of the additivity of the analyzed RT or spectral amplitude measures would indicate synergistic interactions. This hypothesis was tested on both RT and pre-target spectral amplitude measures as described later.

As data followed a normal distribution (determined by Shapiro-Wilk tests), repeated measures analysis of variance (rANOVA) was used to compare the means between groups. Where main effects or interactions were found, statistical results were further specified by post hoc Tukey’s honestly significant difference (HSD). The null hypothesis was rejected at a significance level of 0.05 (alpha). Each P-value with partial eta- squared estimates of effect size are given in the Results Section.

#### Reaction time

We analyzed RT to correctly responded target which occurred between 50 ms and 1000 ms after the stimulus onset^45^. All responses outside this interval, and when the participants made no button press to targets were ignored. Due to the 80% of the acceptable target accuracy level and data loss two participants were excluded from further analysis. The final sample in the RT analysis comprised data from 23 participants (13 female, mean age 20.35 years, SD 1.4). First, possible target probability effects were analyzed on all probability categories (from Prob1 to Prob7) with a two-way rANOVA (Treatment [Control vs. UMTS vs. Caffeine vs. Combined] X Probability [Prob1 to Prob7]). Hereafter, two probability categories (Prob1 as Low and Prob4 as High) were chosen for further analysis. The possible synergistic effects on RT were analyzed with two-way rANOVA (Treatment [Control vs. UMTS vs. Caffeine vs. Combined] X Probability [Low vs. High]). Furthermore, to analyze the possible Treatment effects even more precisely, we divided RT data into Low only and High only categories and applied one-way rANOVA (Treatment [Control vs. UMTS vs. Caffeine vs. Combined]). We also analyzed possible probability effects with Student’s t-test in each Treatment condition separately.

#### Spectral amplitudes in the pre-target period

**Figure 4:**
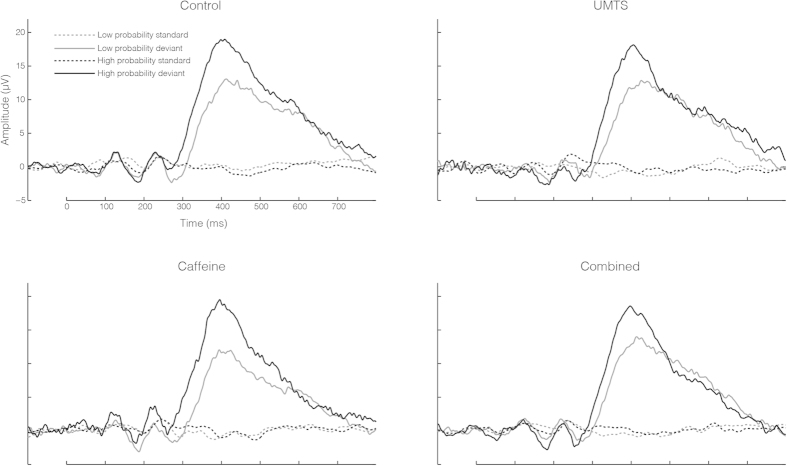
Grand-average event related potentials (ERPs) recorded from Pz electrode site in each treatment (Control, UMTS, Caffeine and Combined). ERPs to standard and deviant stimuli (target) are shown in both Low and High probability categories. The Low and High probability standards were the ERPs evoked immediately before the Low and High probability targets, respectively. Note: for abbreviations see Fig. 2.

Continuous EEG data were off-line band-pass filtered between 0.5 Hz and 80 Hz with 50 Hz notch filter. Pre-target epochs from 600 ms preceding targets to the stimuli onset (0 ms) were extracted. Since mean SOA was 1500 ms, the analyzed the segments, which contained mostly spontaneous activity and were mostly free from activity evoked by the standard stimulus, which preceded the target (Fig. 4). For off-line artifact rejection purposes all epochs exceeding ±100 uV on any of the electrodes including the EOG electrode or epochs containing incorrect behavioral response were excluded from further analysis. The overall mean analyzed trial number was 17.02 (SEM: 0.14) and average acceptance trial rate (analyzed/presented trials*100) was 92% (SEM: +−9). There were significant differences between the analyzed trial numbers across the Probability categories (Low, High) [F(1,20) = 0.001; p = 0.98; partial eta-squared < 0.01] or Treatment (Control, UMTS, Caffeine, Combined) [F(3,60) = 0.34; p = 0.79; partial eta-squared < 0.02].

Due to excessive artifacts or data loss, four participants were excluded from further analysis. The final sample in the spectral amplitude analysis comprised data of 21 participants (11 female, mean age 20.48 years, SD 1.4). Fast Fourier transformation (FFT) was applied on the artifact free, epoched data with 1 Hz resolution to get spectral power values. These values were than transformed with the following formula^24^:


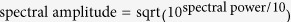


Spectral amplitudes were calculated for pre-defined frequency bands for statistical analysis (alphaI: 8–10 Hz, alphaII: 11–13 Hz, gamma1: 33–46 Hz, gamma2: 54–70 Hz). The possible synergistic effects on the amplitudes of the Alpha1, Alpha2, Gamma1 and Gamma2 frequency bands were analyzed at posterior (P3, P4, O1, O2, P7, P8, Pz, Cp1, Cp2, Cp5, Cp6) electrode sites with three-way rANOVA (Probability X Treatment X Electrode), respectively^46^. Where Treatment or Probability category main effects were found, analyses were further refined by dividing the data into Low/High only probability or Control/UMTS/Caffeine/Combined only subgroups, respectively. On the refined data-sets two-way rANOVA (Treatment X Electrode) or rANOVA (ProbabilityCategory X Electrode) were applied.

#### Multiple regression

Multiple regression statistical method was used to address the question how the different treatments contributed to behavioral effects observed in RT measures controlling for probability categories, and pre-target EEG amplitudes. The interaction test between the Probability and the Treatments allows us to investigate whether or not the caffeine effect was modulated by the target probability. Here we applied the following multiple regression equation:


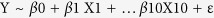


In this equation Y is the predicted variable, X1 … X10 are the predictor variables, *β*0 is the intercept, and ε is the error. The terms *β*1 … *β*10 are the estimated slope parameters, which are used as multipliers for the corresponding X1 … X10 predictor variables and their interactions, respectively.

First, to address the question whether any treatment (UMTS, Caffeine, or Combined) affects the RT, we applied categorical variables on Treatments where Control treatment served as reference also controlling for probabilities and pre-target spectral amplitude as predictor variables. We applied the following multiple regression model:


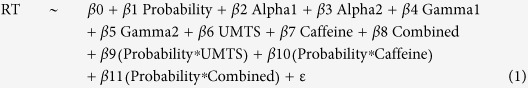


Second, to reveal any potential combined effects of caffeine and UMTS MP exposures we applied the same regression model, with the categorical variable for Combined treatment serving as the reference this time. The model is as follows:


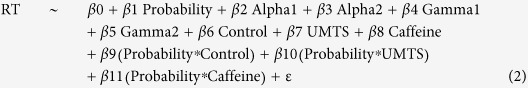


## Results

### Reaction time

Overall, the analysis of the 7 probability categories (Prob1 to Prob7) showed significant main effects of Probability [F(6,132) = 33.615, p < 0.001, eta-squared = 0.604] and Treatment [F(3,66) = 3.035, p = 0.035, eta-squared = 0.12]. The linear-log statistical model [y = a*log(x) + b] showed significantly decreasing (p < 0.001) logarithmic correlation (R^2^ = 0.8832) between the target-target probabilities and the reaction times. Kendall’s correlation also showed that RT marginally decreased (tau = −0.619, p = 0.069) from the Prob1 (mean: 442.22 ms, SEM: 10.16) to Prob7 (mean: 399.17 ms, SEM: 11.15) (Fig. 3b).

The analysis of the Low (mean: 442.22 ms, SEM: 10.16) and High (mean: 398.54 ms, SEM: 9.71) probability categories revealed that they significantly differed from each other [F(1,22) = 102.584, p < 0.001, eta-squared = 0.823]. Furthermore, a significant Treatment main effect [F(3,66) = 2.849, p < 0.045, eta-squared = 0.115] was found. However the Treatment effect did not survive the post hoc analyses.

**Figure 5:**
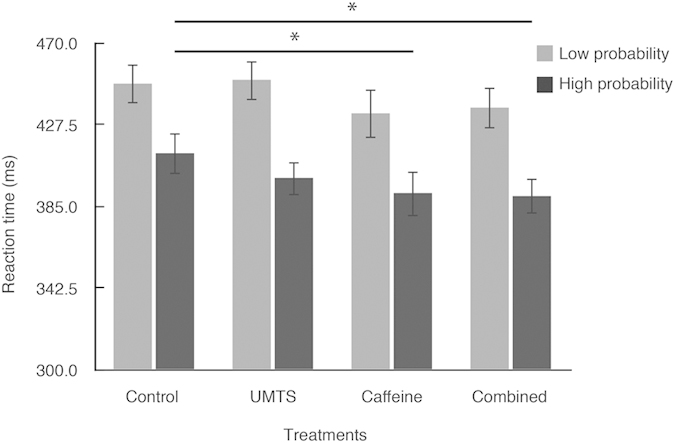
Results for reaction time (RT) to target stimuli in each treatment condition (Control, UMTS, Caffeine, Combined). Caffeine treatment significantly decreased the High probability RT relative to the Control (placebo). We found no combined effects of caffeine and UMTS exposure on RT. Note: *p < 0.05; for abbreviations see Fig. 2.

When we separated RTs into Low only and High only probability categories, the analysis revealed significant Treatment main effects only in the High category [F(3,66) = 3.621, p < 0.018, eta-squared: 0.14]. The Tukey HSD post hoc test indicated that the High RT shortened in the caffeine (p < 0.039) and combined (p < 0.023) treatments compared to control (Fig. 5). The probability effects were analyzed in all Treatments, separately. Student’s t-test showed significant differences between Low and High probability categories in each Treatment (all p < 0.001). The summative analysis did not show any synergistic interactions on the RT.

### Spectral amplitudes in the pre-target period

**Figure 6:**
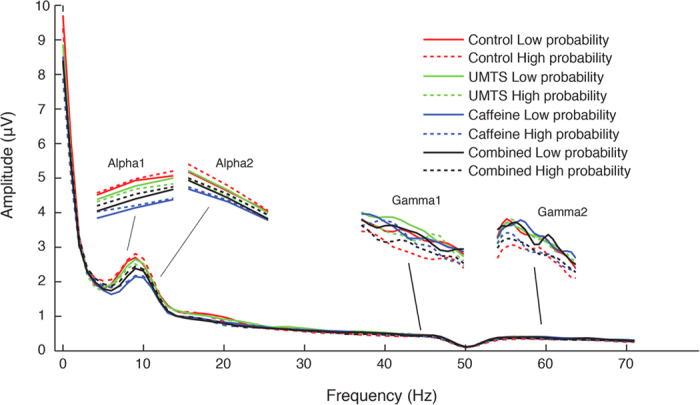
Log-transformed pre-target (−600 to 0 ms) spectral power at the analyzed electrode sites in each treatment and in analyzed each probability category (Low and High). Note: for abbreviations see Fig. 2.

Pre-target EEG amplitudes are shown in Fig. 6.

**Figure 7:**
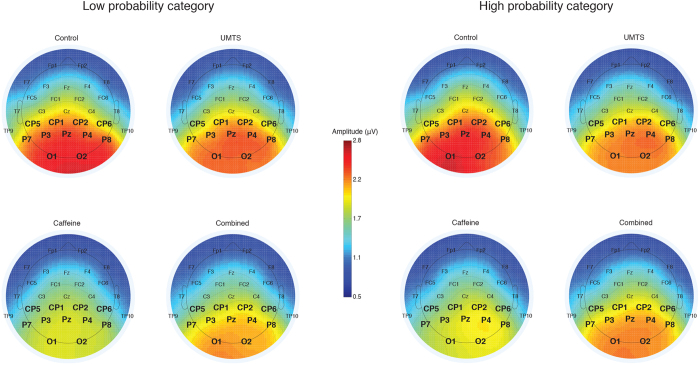
Scalp topographic maps of the Low and High probability Alpha1 amplitudes in each treatment. Colors represent the mean Alpha1 amplitudes in the −600 to 0 ms time period preceding the targets. The electrode sites in the region of interests are marked with bold face. Note: for abbreviations see Fig. 2.

Significant Treatment effects were found in alpha1 [F(3,60) = 8.779, p < 0.005, eta-squared = 0.305] and alpha2 [F(3,60) = 5.552, p < 0.005, eta-squared = 0.217] frequency bands. Tukey HSD post hoc analysis revealed significant amplitude decrease of both alpha bands [alpha1 (p < 0.005), alpha2 (p < 0.005)] in the caffeine treatment compared to control. Furthermore, the alpha1 amplitude significantly decreased in the caffeine and in the combined treatment compared to UMTS (p < 0.005) or control (p < 0.05), respectively. Treatment main effects were further specified by dividing the data into Low and High probability categories. The effects of caffeine on the alpha1 amplitude are shown in Fig. 7.

**Figure 8:**
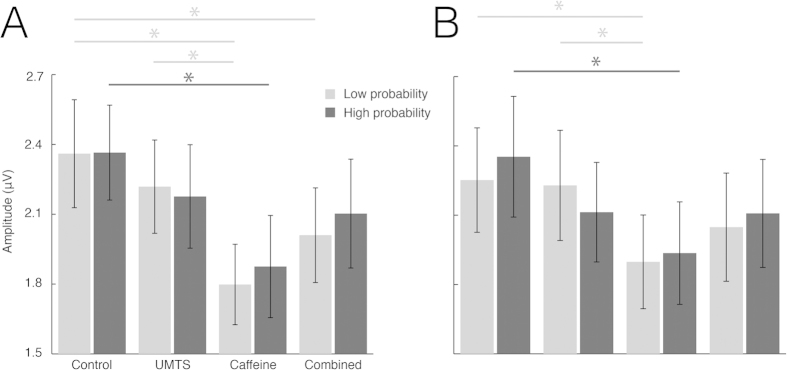
(**A**) Results for Alpha1 amplitudes in each treatment. Caffeine significantly decreased both Low and High probability Alpha1 amplitudes relative to the Control. We found no combined effects of caffeine and UMTS exposure on the Alpha1. (**B**) Results for Alpha2 amplitudes in each treatment. Caffeine significantly decreased the High Alpha2 amplitude relative to the Control. We found no combined effects of caffeine and UMTS exposure on the Alpha2. Note: *p < 0.05; for abbreviations see Fig. 2.

The rANOVA yielded the following significant main effects: Low Alpha1: F(3,60) = 7.852, p < 0.005, eta-squared = 0.282; High Alpha1: F(3,60) = 5.547, p < 0.005, eta-squared = 0.217; Low Alpha2: F(3,60) = 4.398, p < 0.005, eta-squared = 0.180; High Alpha2: F(3,60) = 4.032, p < 0.05, eta-squared = 0.168. The results of the Tukey HSD post hoc analysis are shown in Fig. 8. The rANOVA [(sum vs. simultaneous) X Probability X Electrode] of the summative model indicated no synergistic interactions of caffeine and UMTS exposure on either alpha1 or alpha2 frequency bands.

Significant probability main effects was found in both gamma1 [F(1,20) = 18.731, p < 0.005, eta-squared = 0.484] and gamma2 [F(1,20) = 33.908, p < 0.005, eta-squared = 0.629] bands in the pre-target period. We found that gamma1 and gamma2 pre-target amplitudes were significantly lower in the High probability category (gamma1: mean = 0.518 uV, SEM: 0.025; gamma2: mean = 0.364 uV, SEM = 0.015) than in the Low probability (gamma1: mean = 0.551 uV, SEM: 0.027; gamma2: mean = 0.396 uV, SEM: 0.015). No specific Treatment effects were found. When we divided the data into separate datasets containing only Low and High probability categories, significant probability main effects were found on all models except in caffeine gamma1 and UMTS gamma2 conditions.

The rANOVA [(sum vs. simultaneous) X Probability X Electrode] of the summative model indicated no interactive effects of caffeine and UMTS exposure on either gamma1 or gamma2 frequency bands.

### Multiple regression analysis

Estimated parameters and their significance levels for the Equation 1 and Equation 2 are shown in the Table 1.

## Discussion

In the present study, we investigated possible synergistic effects of caffeine and acute UMTS MP exposure on target local probability indexed by RT and pre-target brain activity in a visual target detection task, where participants discriminated between frequent standard and rare target stimuli and responded with a button-press to the latter. Caffeine exposure also served as pharmacological (positive) control as it has been previously reported that intraoral administration of 3 mg/kg b.w. caffeine reliably improved cognitive functions and mood^7^,^9^. To test the possible synergism we adopted an additive analysis model^43^,^44^.

Reaction time and brain responses are known to show remarkable individual trial by trial variability depending on the actual arousal state or attentional level of the participants^40^. In the present study RTs were highly dependent on the number of standard stimuli preceding the targets, namely RTs significantly decreased for targets with higher local probability (High, preceded by 4 standards) compared to low probability targets (Low, preceded by 1 standard). Our results are in line with findings in numerous previous studies which investigated the effects of perceived distance to target stimuli by the increasing number of preceding non-targets on RTs and event-related potentials^40^,^41^,^47^. Most of them found that RTs and brain responses to infrequent target stimuli negatively correlated with the number of the frequent non-target stimuli preceding that target. In addition, here we found that caffeine further facilitated RT for High local probability targets, whereas it did not improve RT for targets with Low local probability. The present results are in line with previous findings showing that caffeine decreases RT^6^,^48^,^49^ and we also show here that the decreased RT is mainly mediated by responses to highly expected stimuli. One possible explanation of our findings is that caffeine speeds up High RTs, which belong to more overt attention. Thus, it would be reasonable to conclude that caffeine increases the sensitivity to the stimulus pattern, and improves the efficiency of implicit short-term memory^41^. However, the results of the multiple regression models showed no interaction effects between the target Probability and Treatments which support a different possible explanation of the present results. Namely, caffeine only facilitates the faster initiation of an already prepared response, independently of the underlying differential cognitive processes (e.g., higher or lower stimulus expectancy). However, to draw the final conclusion we suggest that future studies with more focused task design should address this question.

In line with the results of previous studies^50^,^51^ we found no evidence that UMTS exposure alters RT. In addition, the present results do not support the notion that UMTS exposure may strengthen the observed facilitatory effects of caffeine on RT in a combined or synergistic manner as results of our additive analysis model did not suggest any interactive effects.

Several studies have investigated the role of the alpha-band oscillation during resting conditions and during cognitive task performance and it is widely accepted that the alpha frequency band is an important readout of ongoing attentional processes^52^,^53^. For example, using a go/no-go task, Foxe *et al.*^24^ found that caffeine decreased alpha power in the pre-target period. In the present study we used a simple visual oddball paradigm and we also found that caffeine decreased the pre-target alpha power both in Low and High target probability conditions. One possible explanation of the decreased alpha band activity in the pre-target by caffeine may be that caffeine increased the arousal state, thus promoted better visual perception performance compared to the control condition^54^. Alternatively, caffeine may have enhanced neural excitability in general^55^. Our results of the multiple regression analysis on alpha1 amplitude and target probability suggest that RT was more influenced by the target probability than by the pre-target alpha amplitude in all non-caffeine conditions. However, in the caffeine condition the RT showed a stronger relationship with the alpha1 amplitude than with target probability. One possible explanation may be that caffeine has a general positive effect on general attentional resource allocation independently of the relative target probability^56^.

Several studies investigated the role of gamma oscillations in various cognitive functions. For example, pre-target gamma activity was found sensitive to top-down attention, especially when participants highly anticipated the occurrence of the target stimulus^32^. Elsewhere, oscillation power in the gamma band reportedly predicted the speed of RT to the forthcoming stimuli. Reinhart and co-workers tested their hypothesis in an auditory paradigm, where participants had to respond to target tones^35^. The authors found that the decrease of gamma power in the pre-target period positively correlated with the RT. In the same study, it was also suggested that higher gamma power indicated more effective response preparation in the pre-target period, as reflected in the more detailed, but much slower evaluation of the forthcoming target stimulus^35^. In another study by Gómez and co-workers the authors found a generalized decrease in the oscillatory activity in the pre-target period, and suggested that reduction of the gamma power speeds up the processing of the forthcoming target stimulus^36^. In line with their results, in the present study, in the pre-target period, we found lower gamma amplitude with faster RT in the High local probability condition and higher gamma amplitude with slower RT in the Low local probability condition. Thus, it is likely that the decreased gamma activity prior to the predicted arrival of a target stimulus may facilitate the processing of relevant task-related information^36^.

The present results showing no effect of UMTS MP-like exposure on the alpha and gamma power in the pre-target period correspond well with previous studies using resting EEG, where also no effects of UMTS exposure were reported on brain oscillations^2^,^37^,^57^,^58^. Thus, our results further support the notion that an acute, 15 min exposure to UMTS MP-like EMF signal alone does not affect neural activity concerning decision making in a visual discrimination task. Furthermore, we found no evidence of any interaction between caffeine and UMTS exposure on either RT or brain oscillations (spectral amplitude) using rANOVA or the additive analysis models. In addition, we suggest that 15 min UMTS MP-like EMF exposure does not influence (increase or decrease) the observed facilitatory effects of caffeine on behavioral or electrophysiological measures of cognitive performance in a visual discrimination task. These null effects of UMTS exposure on visual discrimination are in line our previous report on the same behavioral dataset^5^ where we found that the UMTS exposure had no observable modulatory effects on RT and P300 ERP measures either alone or in combination with caffeine.

## Conclusion

We found that caffeine speeds up responses to highly expected targets and facilitates allocation of attentional resources as indexed changes in pre-target alpha amplitude. Furthermore, pre-target gamma amplitude negatively correlated with target probability. However, no effects of UMTS exposure were observed alone or in combination with caffeine, suggesting that UMTS exposure did not have any additional facilitatory effect on visual target detection. A possible explanation for lack of UMTS exposure effects may be that the applied signal modulation was ineffective or the signal intensity was too low, i.e., under the threshold for detectable biological effects. However, at this point, it cannot be generally ruled out, as also suggested by Juutilainen *et al.*^59^, that other types of frequently used EMF modulation may exceed the threshold for biological effects, either alone or in combination with chemical or other agents (e.g., combined modulation type of EMF). As in the present study and in our previous report^5^ we also applied positive pharmacological manipulation to explore possible additive effects of UMTS exposure on known facilitatory brain activation in a full factorial recording design, we generally conclude that an acute 15 min UMTS exposure does not alter RT or pre-target oscillatory activity.

## Additional Information

**How to cite this article**: Trunk, A. *et al.* Effects of concurrent caffeine and mobile phone exposure on local target probability processing in the human brain. *Sci. Rep.* **5**, 14434; doi: 10.1038/srep14434 (2015).

## Figures and Tables

**Table 1:** Results for the multiple regression analysis.

Parameters	Equation 1			Equation 2		
	Predictor variables	Estimated parameters	Significance levels	Predictor variables	Estimated parameters	Significance levels
*β*0	Intercept	257.69	<0.05	Intercept	229.21	<0.05
*β*1	Probability	−3.77	0.09	Probability	−4.33	<0.05
*β*2	Alpha1	−17.8	NS	Alpha1	−17.8	NS
*β*3	Alpha2	−20.74	NS	Alpha2	−20.74	NS
*β*4	Gamma1	565.54	<0.05	Gamma1	565.54	<0.05
*β*5	Gamma2	−12.69	NS	Gamma2	−12.69	NS
*β*6	UMTS	−14.98	NS	Control	28.47	0.08
*β*7	Caffeine	−34.5	0.08	UMTS	13.49	NS
*β*8	Combined	−28.47	0.08	Caffeine	−6.03	NS
*β*9	Probability*UMTS	−1.92	NS	Probability*Control	0.57	NS
*β*10	Probability*Caffeine	−2.41	NS	Probability*UMTS	−1.36	NS
*β*11	Probability*Combined	−0.57	NS	Probability*Caffeine	−1.85	NS

Estimated parameters and significance levels with predictor variables are listed for Equation 1 and Equation 2, respectively. In the Equation 1 and Equation 2, Control and Combined treatments were used as reference variables, respectively.
